# Sinus Microanatomy and Microbiota in a Rabbit Model of Rhinosinusitis

**DOI:** 10.3389/fcimb.2017.00540

**Published:** 2018-01-12

**Authors:** Do-Yeon Cho, Calvin Mackey, William J. Van Der Pol, Daniel Skinner, Casey D. Morrow, Trenton R. Schoeb, Steven M. Rowe, William E. Swords, Guillermo J. Tearney, Bradford A. Woodworth

**Affiliations:** ^1^Department of Otolaryngology Head and Neck Surgery, University of Alabama at Birmingham, Birmingham, AL, United States; ^2^Gregory Fleming James Cystic Fibrosis Research Center, University of Alabama at Birmingham, Birmingham, AL, United States; ^3^Center for Clinical and Translational Science–Informatics, University of Alabama at Birmingham, Birmingham, AL, United States; ^4^Departments of Cell, Developmental and Integrative Biology, University of Alabama at Birmingham, Birmingham, AL, United States; ^5^Departments of Medicine, Pediatrics, Cell Developmental and Integrative Biology, University of Alabama at Birmingham, Birmingham, AL, United States; ^6^Departments of Medicine, Microbiology, University of Alabama at Birmingham, Birmingham, AL, United States; ^7^Wellman Center for Photomedicine, Massachusetts General Hospital, Boston, MA, United States; ^8^Department of Pathology, Harvard Medical School, Boston, MA, United States

**Keywords:** airsurface liquid, microbiome, microbiota, dysbiosis, chronic rhinosinusitis, rhinosinusitis, rabbit model, animal model

## Abstract

**Background:** Rabbits are useful for preclinical studies of sinusitis because of similar physiologic features to humans. The objective of this study is to develop a rabbit model of sinusitis that permits assessment of microanatomy and sampling for evaluating shifts in the sinus microbiota during the development of sinusitis and to test how the mucociliary clearance (MCC) defect might lead to dysbiosis and chronic rhinosinusitis (CRS).

**Methods:** Generation of CRS was accomplished with an insertion of a sterile sponge into the left middle meatus of New Zealand white rabbits (*n* = 9) for 2 weeks. After sponge removal, 4 rabbits were observed for another 10 weeks and evaluated for CRS using endoscopy, microCT, visualization of the functional micro-anatomy by micro-optical coherence tomography (μOCT), and histopathological analysis of the sinus mucosa. Samples were taken from the left middle meatus and submitted for microbiome analysis.

**Results:** CT demonstrated opacification of all left sinuses at 2 weeks in all rabbits (*n* = 9), which persisted in animals followed for another 12 weeks (*n* = 4). Histology at week 2 showed mostly neutrophils. On week 14, significant infiltration of plasma cells and lymphocytes was noted with increased submucosal glands compared to controls (*p* = 0.02). Functional microanatomy at 2 weeks showed diminished periciliary layer (PCL) depth (*p* < 0.0001) and mucus transport (*p* = 0.0044) compared to controls despite a thick mucus layer. By 12 weeks, the thickened mucus layer was resolved but PCL depletion persisted in addition to decreased ciliary beat frequency (CBF; *p* < 0.0001). The mucin fermenting microbes (*Lactobacillales, Bacteroidales*) dominated on week 2 and there was a significant shift to potential pathogens (e.g., *Pseudomonas, Burkholderia*) by week 14 compared to both controls and the acute phase (*p* < 0.05).

**Conclusion:** We anticipate this reproducible model will provide a means for identifying underlying mechanisms of airway-surface liquid (ASL) depletion and fundamental changes in sinus microbial communities that contribute to the development of CRS. The rabbit model of sinusitis exhibited diminished PCL depth with delayed mucus transport and significant alterations and shift in the sinus microbiome during the development of chronic inflammation.

## Introduction

The normal sinonasal mucociliary function is a critical host defense mechanism that clears the inhaled particles such as bacteria, dust, and aerosols. Characterized by impaired mucociliary clearance (MCC) with subsequent compromised microbial elimination, chronic rhinosinusitis (CRS) is known as a multifactorial and idiosyncratic disease process in which bacterial infection or colonization may play some role in initiating or sustaining the inflammatory response (Ramakrishnan et al., 2013; Woodworth, 2015). Disruption of commensal microbiota (dysbiosis) can lead to benign microbial communities becoming pro-inflammatory, leading to invasion or overgrowth of pathogens (Dickson et al., 2014). There is growing evidence that dysbiosis of the sinus microbiota is associated with CRS pathogenesis (Orlandi et al., 2016). Recent studies have revealed that the microbiome of CRS is characterized by less richness, evenness, and diversity compared to healthy controls (Yan et al., 2013; Wilson and Hamilos, 2014). However, studies of human sinus microbiota are especially challenging because medical therapies (e.g., topical or systemic antibiotic administration) can dramatically decrease the richness and diversity of resident bacterial communities (Dethlefsen et al., 2008; Liu C. M. et al., 2013; Orlandi et al., 2016). Observed alterations in local microbiota in human CRS may result from repeated and prolonged medical therapies (Liu et al., 2015). Thus, it is unclear how dysbiosis develops from normal sinus and how shifts in bacterial communities might contribute to CRS development.

To answer these questions, a preclinical model of sinusitis is critical for longitudinal sampling to determine microbiome stability and resilience to perturbation, as well as mucociliary function. The immunologic features of rabbit and human sinonasal epithelium are similar (Vaure and Liu, 2014), and the *in vivo* rabbit acute sinusitis disease model has already been established and shown to be well-suited for studies of therapeutic intervention (Chiu et al., 2007). However, limitations of previous rabbit models include traumatic disruption of mucoperiosteum during surgery and inoculation with pathogenic bacteria, which generate additional variables that may influence changes in mucociliary transport. To study changes in the rabbit sinus microbiome and mucociliary function, blockage of the sinus ostium must be sterile and atraumatic to the sinus cavity. Transient, atraumatic blockade of the sinus ostia (MCC defect) for 2 weeks is critical to evaluate the effects of MCC dysfunction on the microbiota and how dysbiosis might perpetuate persistent defects in mucus transport leading to the CRS disease phenotype using this animal model. The objectives of the current study are to (1) develop a rabbit model of sinusitis that permits assessment of microanatomy (mucociliary function) and sampling for evaluating shifts in the sinus microbiota during the development of sinusitis and (2) test how the MCC defect might lead to dysbiosis and CRS.

## Methods

### Animal model

This study was approved by the Institutional Animal Care and Use Committee (IACUC) at the University of Alabama at Birmingham (UAB). *Pasteurella*-free, female, New Zealand white rabbits (2–4 kg) were used for the study. Before initiation, rabbits were acclimatized to the animal facility for at least 1 week. For any procedure, rabbits were anesthetized with [ketamine (20 mg/kg) (MWI, Boise, ID), dextomitor (0.25 mg/kg) (Zoetis Inc., Kalamazoo, MI), buprenorphine (0.03 mg/kg) (Reckitt Benckiser Pharmaceuticals Inc., Richmond, VA), and carprofen (5 mg/kg) (Zoetis Inc., Kalamazoo, MI)] in a warm room for comfort. Rabbits did not receive any antibiotics before or during this study.

### Rabbit model of sinusitis

**Figure 1:**
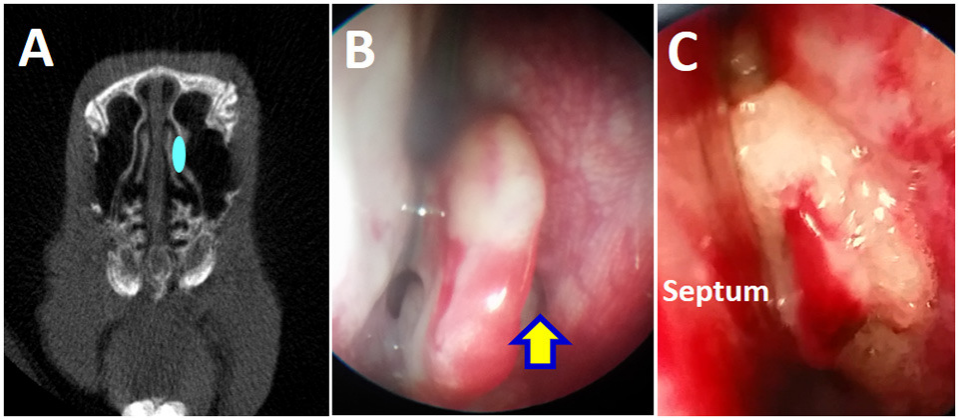
Placement of sterile sponge in the left middle meatus. **(A)** Schematic drawing of sponge placement in the rabbit's left middle meatus to block the maxillary sinus drainage pathway. **(B)** Endoscopic view of left middle meatus (yellow arrow). **(C)** Placement of sponge in the left middle meatus.

A total of 9 rabbits were used to create a rabbit model of sinusitis without providing exogenous bacteria or pathogens. A sterile synthetic sponge (Merocel®, Medtronic, Minneapolis, MN) was placed in the left (unilateral) middle meatus (natural outflow tract of maxillary sinus) of New Zealand white rabbits for 2 weeks (Figure 1). This method (transnasal endoscopic approach) causes no damage to the sinus mucosa and is reversible by removing the sponge transnasally on week 2. On week 2, synthetic sponges were removed from left middle meatus in all nine rabbits. After removal, 5 rabbits were assessed for the development of acute rhinosinusitis (ARS) and the other 4 rabbits were simply observed for the next 12 weeks to examine whether these rabbits developed CRS features. At each time point (Day 0, Week 2, and Week 14), rabbits were examined with micro-computed tomography (microCT) scanning, nasal endoscopy, microbiome analysis, micro-optical coherence tomography (μOCT), and histopathology.

1. Micro-Computed Tomography (CT) scanning—All rabbits were scanned before the placement of the sponge on Day 0. CT scanning was repeated on Week 2 (*n* = 9) and Week 14 (*n* = 4). Micro CT scanning was performed at the UAB small animal imaging shared facility using SPECT/CT (X-SPECT system, Gamma Medica, Northridge, CA). CT findings of sinus opacification were scored as follows: 1: mild; 2: moderate; 3: severe; 4: very severe, based on previous radiological grading methods in experimental rabbit sinusitis (Ozcan et al., 2011). An absence of any opacification was scored as 0.
2. Nasal endoscopic examination—A 1.7 mm 30-degree endoscope (Karl Storz, Tuttlingen, Germany), was used to examine the nasal cavity on Day 0 (*n* = 9), Week 2 (*n* = 9), and Week 14 (*n* = 4) as explained previously (Cho et al., 2016a).
3. Micro-optical coherence tomography (μOCT) image acquisition and analysis—Rabbits were euthanized, and sinus cavities were harvested. Measurements of functional microanatomic parameters in *ex vivo* sinus tissue were performed using μOCT, a high-speed, high-resolution microscopic reflectance imaging modality, according to the protocol outlined by Liu L. et al. (2013). In brief, the μOCT instrument provides cross-sectional images of the airway epithelium at a resolution of ~1 μm. This resolution is sufficient to directly visualize and quantify microanatomic parameters, including periciliary layer (PCL) depth, ciliary beat frequency (CBF), and mucociliary transport (MCT). Typical acquisition speed is 20,480 Hz line rate, resulting in 40 frames/s at 512 lines/frame. Quantitative analysis of images provided several metrics. PCL depths were characterized directly by geometric measurement of the respective layers in Image J software. For PCL, measurement time averaging of images over several frames captured the length of fully extended cilia. CBF was quantified by Fourier analysis of the reflectance of beating cilia using custom code in Matlab (Mathworks, Natick, MA). For maxillary sinus tissue, images were acquired on the relatively flat mucosal surface of the medial and lateral wall with the optical beam scanned. To quantify MCT rate, a line was drawn through the mucus layer and parallel to its direction of transport, and the intensity along this line as a function of time was projected as a 2D image (Chu et al., 2016).
4. Histologic evaluation—Once rabbits were euthanized, heads were harvested for histologic sectioning. The maxillary sinus was not opened to avoid injury to the sinus mucosa. Heads were divided to submit the right and left maxillary sinus separately. Specimens were provided to the UAB Comparative Pathology Laboratory (CPL) for sectioning and evaluation. Ten representative sections of the maxillary sinus were selected and stained with hematoxylin and eosin (H&E). Slides were evaluated by a veterinary pathologist blinded to the specimen and side. Periodic Acid–Schiff (PAS) staining was performed to assess the submucosal glands and random sites were selected to count the number of glands.

### Microbiome analysis

#### Sample acquisition

Middle meatus mucus was collected using a flexible tube under endoscopic guidance to sample in all rabbits. The right middle meatus was not sampled due to possible cross-contamination. These middle meatus samples were processed according to the protocol outlined by Kumar et al. (2014). Briefly, samples were collected and stored at −80°C until analysis. Each sample was dissolved in a buffer solution and then processed with a DNA Miniprep Kit to obtain isolated DNA.

#### Multiplexed 16S amplicon sequencing on the MiSeq® system

Polymerase chain was used to amplify the V4 region of the 16S rRNA gene using primers described by Caporaso et al. (2011). Cycling conditions for the PCR reactions were as follows: initial denature 94°C for 1 min followed by 32 cycles of 94°C for 30 s, 50°C for 1 min, 65°C for 1 min, and a final extension of 65°C for 3 min. The entire PCR reaction was electrophoresed on a 1.0% agarose/Tris-borate-EDTA gel. The PCR product (~380 base pairs) was visualized by UV illumination. The band was excised and purified from the agarose using QIAquick Gel Extraction Kit from Qiagen (Venlo, Netherlands) according to the manufacturer's instructions. The PCR products were then sequenced using the Illumina MiSeq platform (Kozich et al., 2013; Kumar et al., 2014). Paired-end reads of ~250 bp from the V4 region of 16S rDNA were analyzed. The samples were first quantitated using Pico Green, adjusted to a concentration of 4 nM then used for sequencing on the Illumina MiSeq (Kumar et al., 2014). Fastq conversion of the raw data files was performed following de-multiplexing. Quality control of the fastq files was performed which was then subject to quality assessment and filtering using the FASTX toolkit (FASTX). The remainder of the steps was performed using the Quantitative Insight into Microbial Ecology (QIIME) suite, version 1.8 (Lozupone et al., 2007; Navas-Molina et al., 2013; Kumar et al., 2014).

#### Sequence data analysis and composition

The sequence data covered the 16S rRNA V4 region with a PCR product length of ~255 bases and 250 base paired-end reads. Since the overlap between fragments was ~245 bases, the information from both ends of the paired reads was merged to generate a single high quality read using the module “fastq mergepairs” of USEARCH (Edgar, 2010). Read pairs with an overlap of <50 bases or with too many mismatches (>20) in the overlapping region were discarded. Chimeric sequences were also filtered using the “identify_chimeric_seqs.py” module of USEARCH (Edgar, 2010). Overall read quality was assessed before and after filtering using FASTQC. The QIIME data analysis package was used for subsequent 16S rRNA data analysis (Caporaso et al., 2010b). Sequences were grouped into operational taxonomic units (OTUs) using the clustering program UCLUST at a similarity threshold of 0.97% (Edgar, 2010). The Ribosomal Database Program (RDP) classifier was used to make taxonomic assignments (to the genus and/or species level) for all OTUs at confidence threshold of 80% (0.8) (Wang et al., 2007). The RDP classifier was trained using the Greengenes (v13_8) 16S rRNA database (McDonald et al., 2012). The resulting OTU table included all OTUs, their taxonomic identification, and abundance information. OTUs whose average abundance was <0.0005% were filtered out. OTUs were then grouped together to summarize taxon abundance at different hierarchical levels of classification (e.g., phylum, class, order, family, genus, and species). Multiple sequence alignment of OTUs was performed with PyNAST (Caporaso et al., 2010a).

#### Statistics of microbiome

Alpha diversity (diversity within the samples) was calculated using Shannon's diversity matrix which measures both richness (number of OTUs/species present in a sample) and evenness (relative abundance of different OTUs/species and their even distribution in a sample) (Jost, 2007), as implemented in QIIME (Caporaso et al., 2010b). Beta diversity (diversity between the samples) was measured using unweighted Unifrac analysis, as measured by *R*^2^, using permutational multivariate analysis of variance (PERMANOVA) (Lozupone et al., 2006). Principal coordinates analysis (PCoA) was performed by QIIME to visualize the dissimilarity matrix between all samples [control, Week 2 (acute), and Week 14 (chronic)], such that samples that were more similar were closer in space than samples that were more divergent (Tyler et al., 2014). A 3-dimensional PCoA plot was generated using EMPEROR (Vázquez-Baeza et al., 2013). Taxa level analyses (inputs from a rarefied OTU table) were performed in QIIME using Kruskal–Wallis testing with false discovery rate (FDR) correction. The threshold of statistical significance for all tests performed was defined as two-sided *p* ≤ 0.05.

### Statistical analysis

Statistical analyses were conducted using Excel 2016 and GraphPad Prism 6.0 software (La Jolla, Ca) with significance set at *P* < 0.05. Statistical evaluation utilized unpaired Student *t*-tests. Data were expressed ± standard error of the mean.

## Results

### Rabbit model of sinusitis

**Figure 2:**
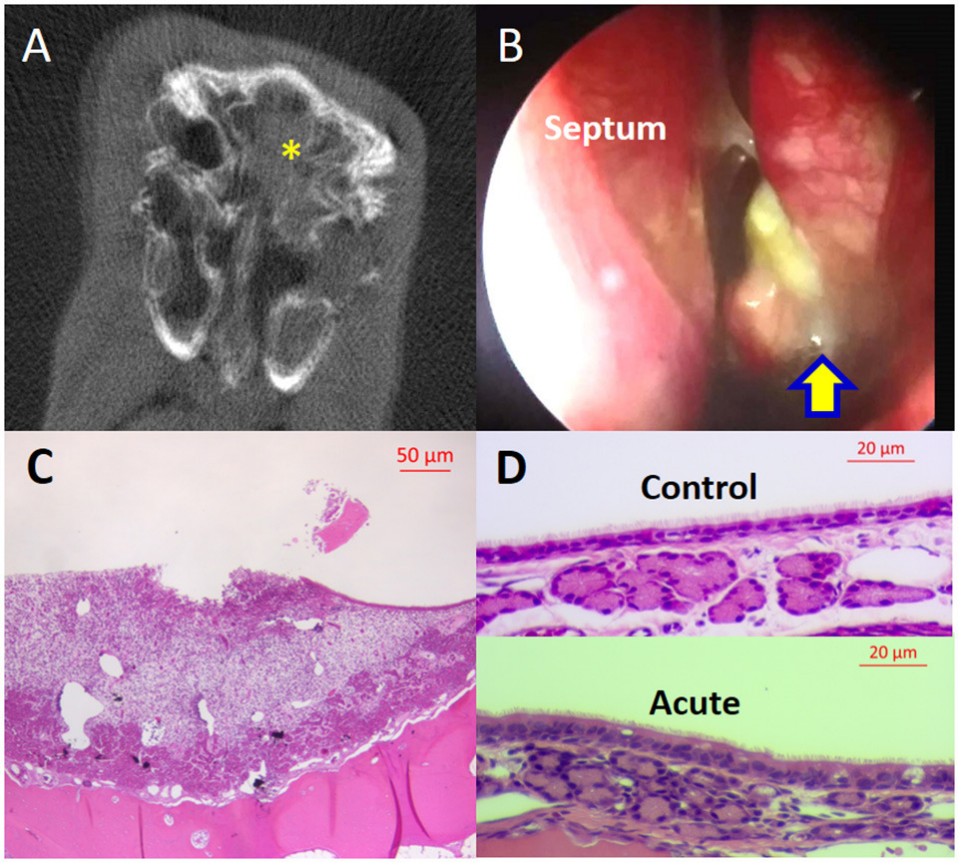
Rabbit model of acute sinusitis (Week 2). **(A)** MicroCT—Full opacification (yellow asterisk) of left sinuses. **(B)** Nasal endoscopic examination—significant purulent drainage from left middle meatus (yellow arrow). **(C)** Histology—superficial mucosal ulceration with infiltration of neutrophils, subepithelial edema, and epithelial hypertrophy (scale bar upper right). **(D)** Histology—upper panel: normal sinus epithelium; lower panel: significant infiltration of inflammatory cells in the epithelium and subepithelial space (scale bar upper right).

**Figure 3:**
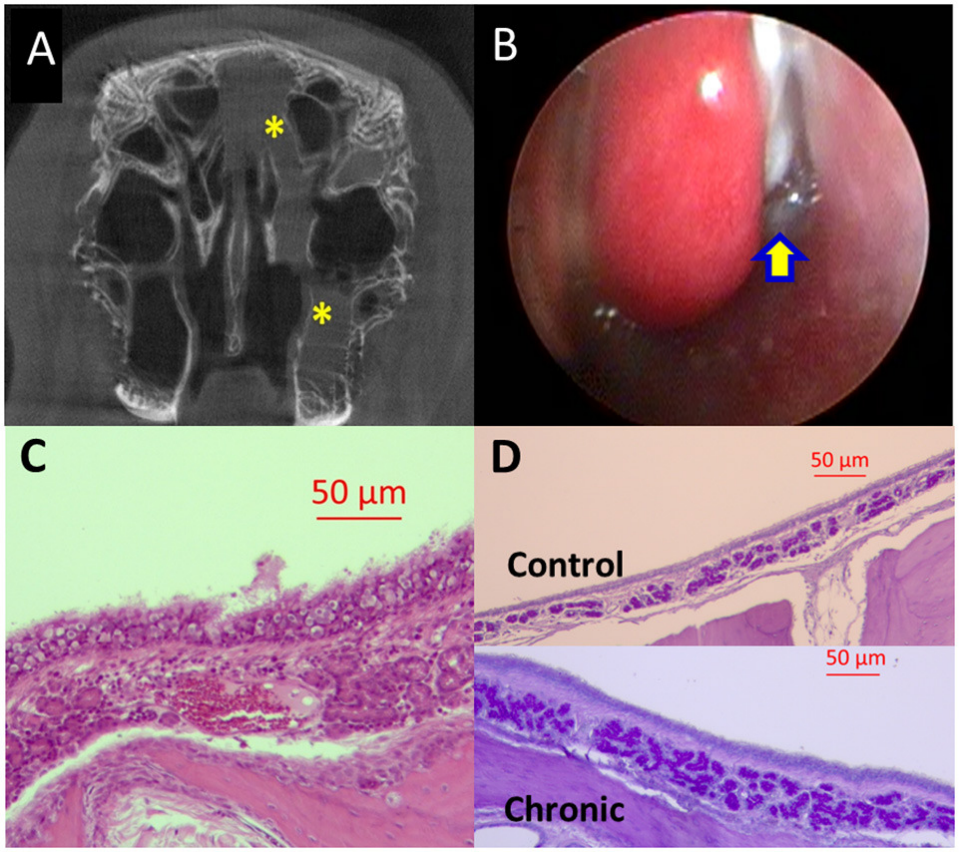
Rabbit model of chronic sinusitis (Week 14). **(A)** MicroCT-partial opacification (yellow asterisk) of left sinuses. **(B)** Nasal endoscopic examination—purulent secretions (yellow arrow) in the middle meatus between middle turbinate and lateral nasal wall. **(C)** Histology—significant infiltration of plasma cells and lymphocytes with inflammatory epithelial hyperplasia and submucosal edema (scale bar upper right). **(D)** Histology—Periodic Acid–Schiff (PAS) staining demonstrated increased submucous glandular density with hypertrophy (lower panel) compared to control (upper panel) (scale bar upper right).

Two weeks after sponge insertion, CT demonstrated opacification of the left sinuses and significant purulent drainage was noticed in the middle meatus in all rabbits (*n* = 9) (Figures 2A,B). Rabbit histology on week 2 showed findings of acute inflammation: significant infiltration with neutrophils and superficial ulceration with sub-epithelial edema was noted (Figures 2C,D). On week 14, all rabbits exhibited opacification of the left sinuses on micro CT scans and had purulent secretions in the middle meatus with scar changes between the middle turbinate and lateral nasal wall on nasal endoscopy (Figures 3A,B). Rabbits demonstrated significantly higher CT scores at week 2 and week 14 compared to day 0 [day 0 = 0 ± 0.00 (*n* = 9), week 2 = 3.56 ± 0.18, week 14 = 2.25 ± 0.48, *p* < 0.0001, Kruskal–Wallis test]. Histologically, significant infiltration of plasma cells and lymphocytes was noted with inflammatory epithelial hyperplasia on week 14 in all rabbits (*n* = 4; Figure 3C). In addition, PAS staining demonstrated increased submucous glandular density with hypertrophy compared to control (Figure 3D). When the numbers of submucous glands were counted at a magnification of × 100 (*n* = 4), a significant increase was noticed when compared to control sides [control = 45.3 ± 2.7, Week 14 = 71 ± 6.5, *p* = 0.02], indicating mucus hyperplasia. Therefore, rabbits exhibited features of acute rhinosinusitis (ARS: edema and extensive infiltration of neutrophils) on week 2 and those of CRS (submucosal gland hyperplasia with infiltration of lymphocytes) on week 14 (Berger et al., 2000; Wu et al., 2011).

### μOCT images

**Figure 4:**
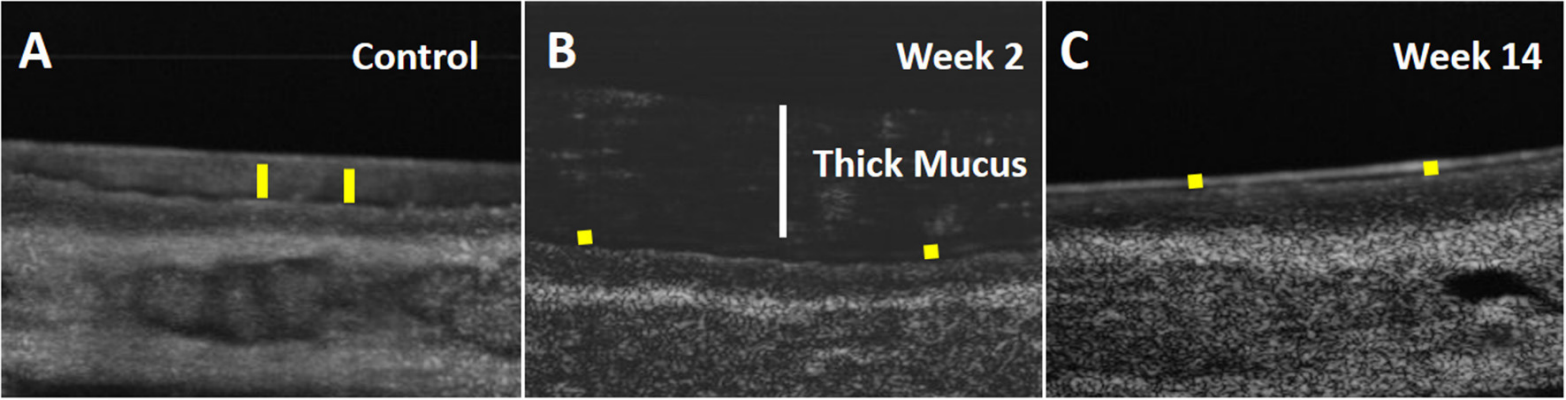
Micro-Optical Coherence Tomography (μOCT) images of *ex vivo* sinus tissue. Time-averaged (1 s) μOCT images of rabbit sinus epithelium. **(A)** Control. **(B)** Week 2 (Acute: White bar represents the thickness of thick mucus). **(C)** Week 14 (Chronic). Yellow bar indicates periciliary liquid (PCL) depth.

**Figure 5:**
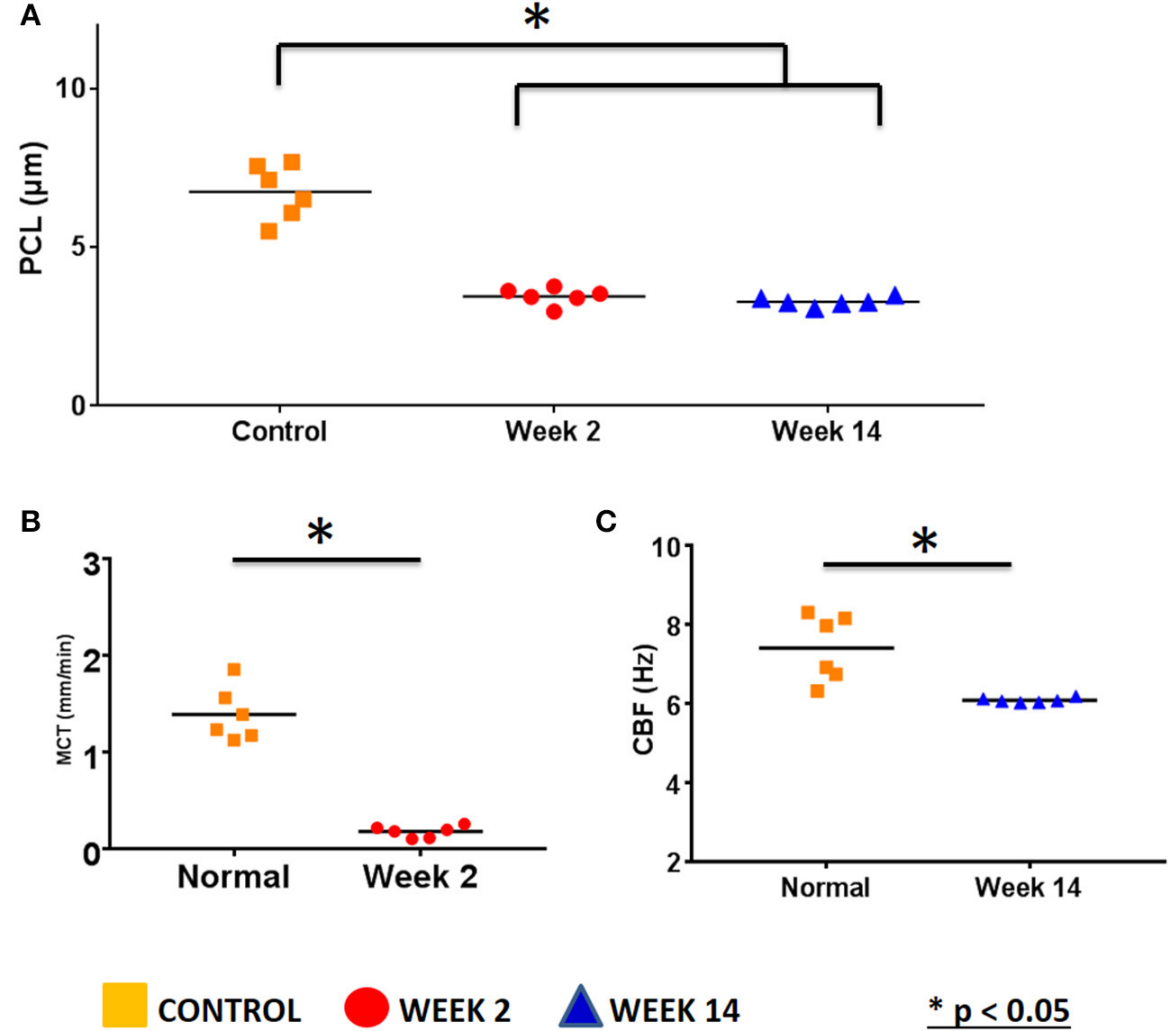
μOCT measurements of *ex vivo* sinus tissue. **(A)** ASL depth measured with μOCT (Control = 6.74 ± 0.4 μm, Week 2 = 3.45 ± 0.1 μm, Week 14 = 3.27 ± 0.06 μm, *p* < 0.0001). **(B)** MCT velocity measured with μOCT (Control = 1.39 ± 0.11 mm/min, Week 2 = 0.18 ± 0.02 mm/min, *p* = 0.0044). **(C)** CBF measured with μOCT (control = 7.4 ± 0.34 Hz vs. Week 14 = 6.08 ± 0.02 Hz, *p* < 0.0001) (^*^P < 0.05).

Respiratory epithelium regulates the depth of the air-surface liquid (ASL) and periciliary (PCL) layers and the viscoelastic properties of the mucus. A high-resolution form of OCT (μOCT) was used to investigate the functional microanatomy of airway epithelia *ex vivo* (Liu L. et al., 2013). Medial and lateral walls of maxillary sinuses from the rabbits (*n* = 6 in each group) were harvested and PCL depth, CBF, and MCT were measured (Figures 4, 5). Maxillary sinus epithelia from both rabbits [week 2 (acute) and week 14 (chronic)] demonstrated significantly lower PCL depth (Week 2 = 3.45 ± 0.1 μm, Week 14 = 3.27 ± 0.06 μm) compared to controls (control = 6.74 ± 0.4 μm) (*p* < 0.0001, Figures 4, 5A). In controls, the mucus was transported quickly by fast-beating cilia without accumulation (MCT velocity = 1.39 ± 0.11 mm/min). On week 2, a thick slow-moving mucus blanket was readily observed (MCT velocity = 0.18 ± 0.02 mm/min, *p* = 0.0044) accompanying the depleted PCL (Figure 5B); CBF was not detectable due to the thickness of the PCL layer. By 14 weeks, the overlying thick mucus layer of the ASL had resolved (Figure 4C), but PCL remained depleted 3.27 ± 0.06 μm, *p* < 0.0001), suggesting residual deficits on anion secretion may exist, as has been shown previously (Cho et al., 2009, 2016b; Cohen et al., 2009; Dransfield et al., 2013; Liu L. et al., 2013; Raju et al., 2013, 2017a,b; Illing et al., 2015; Woodworth, 2015; Cho and Woodworth, 2016; Tipirneni et al., 2017). Consequently, CBF was significantly diminished (control = 7.4 ± 0.34 Hz vs. Week 14 = 6.08 ± 0.02 Hz, *p* < 0.0001) (Figure 5C); MCT was not detectable due to depleted ASL (no traceable mucus), although is likely impaired given the changes in CBF. These data indicate that depleted PCL and aberrant MCT are key aspects of the onset of acute sinusitis, and do not completely resolve as the disease becomes chronic in nature.

### Microbiome

To examine the mechanisms that enable acute sinusitis to transition to a chronic sinusitis phenotype, we hypothesized a shift in the microbiome may be occurring, which could perpetuate the abnormalities. Of the 14 samples examined, a total read count of 588,902 (representing 147,225,500 total base pairs) was obtained, with a mean of 42,064 reads per sample (representing 10,516,000 base pairs/sample, median = 29,963 reads per sample).

**Figure 6:**
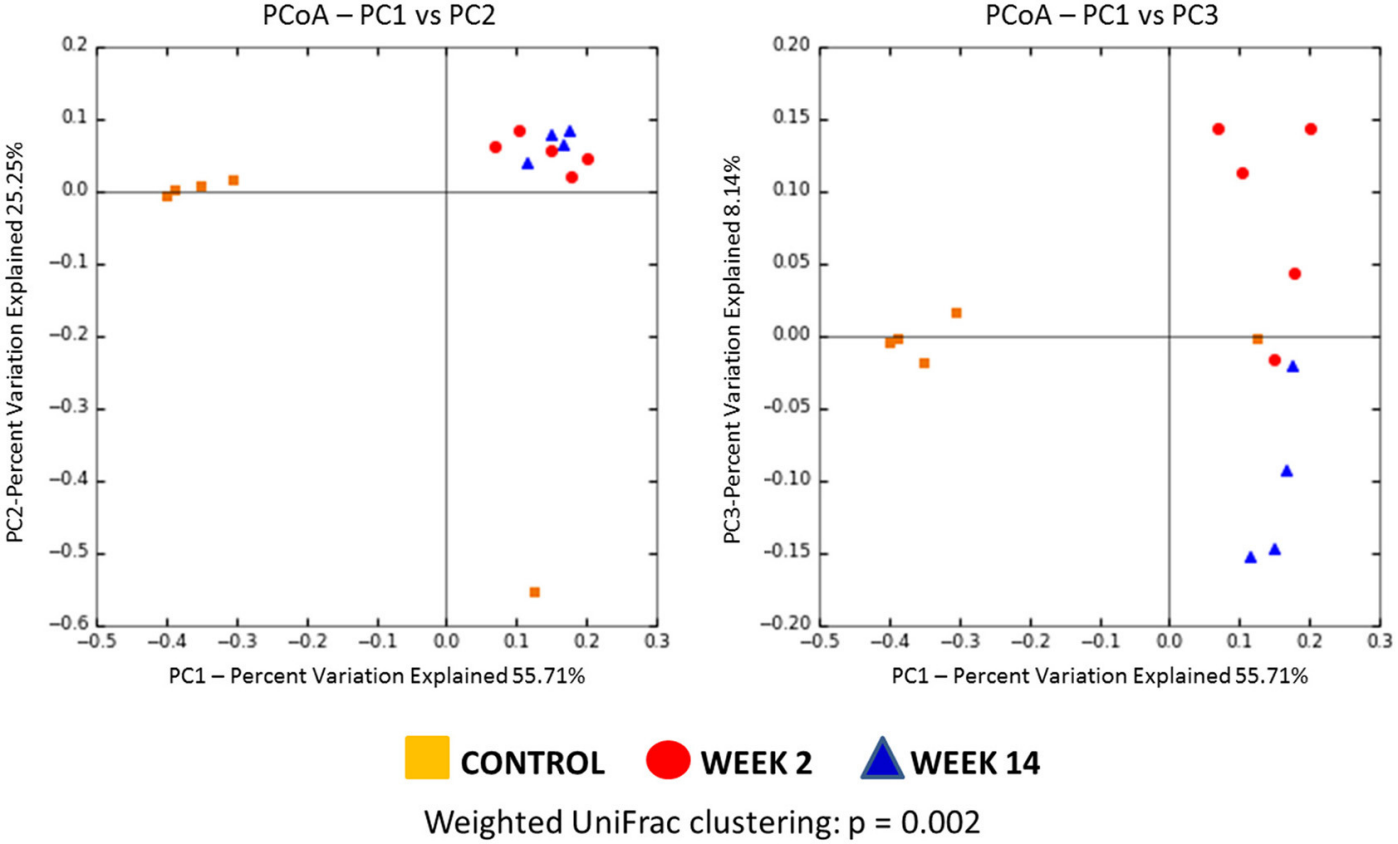
Principal coordinate analysis (PCoA) plot of the microbiome from three groups (Control, Week 2, and Week 14) using weighted UniFrac clustering (Control = Orange, Week 2 = Red, Week 14 = Blue). Using weighted UniFrac clustering, significant differences in beta diversity were found among three groups (*p* = 0.002).

We first assessed the beta diversity to compare these 3 groups [control (day 0), week 2, and week 14], which is an indicator of diversity in the microbial community among these groups. Using weighted UniFrac clustering, unweighted UniFrac clustering and Bray-Curtis similarity index, significant differences in beta diversity were found among the three groups (*p* = 0.002, 0.005, 0.002, respectively). Weighted Unifrac PCoA was used to compare community phylogenetic composition and showed striking microbial diversity clustering among groups (Figure 6).

**Figure 7:**
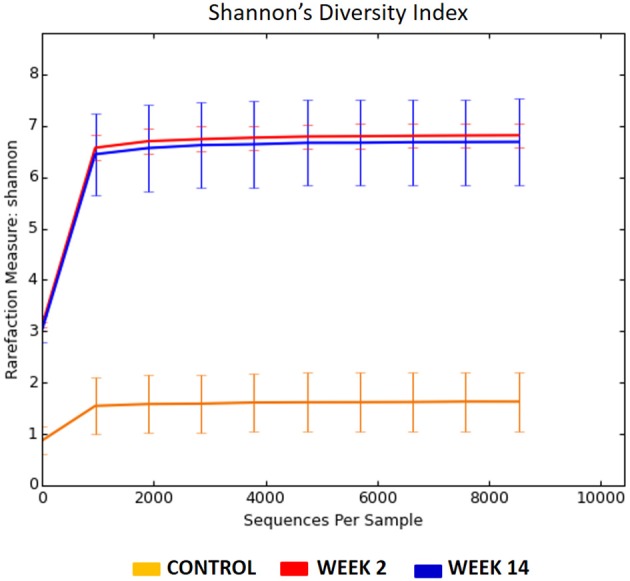
Rarefaction curves for the 16s rRNA gene sequences from three different groups (Control = Orange, Week 2 = Red, Week 14 = Blue). The Shannon's diversity index was significantly elevated in rabbits with acute (week 2) and chronic (week 14) sinusitis compared to controls (Control = 15.34 ± 0.07, Week 2 = 63.93 ± 0.34, Week 14 = 62.71 ± 0.34, *p* < 0.0001).

Then we measured the diversity *within* each group–an indicator of species richness within a single microbial environment. The Shannon's diversity index was significantly elevated in rabbits with acute (week 2) and chronic (week 14) sinusitis compared to controls [Control (day 0) = 15.34 ± 0.07, Week 2 = 63.93 ± 0.34, Week 14 = 62.71 ± 0.34, *p* < 0.0001]. The rarefaction analysis, which represents the number of species present in a given sample, revealed that sequencing effort was sufficient to capture the bacterial diversity of samples (Figure 7). These diversity analysis tools showed that the sinusitis model exhibited higher bacterial diversity compared to their healthy controls (day 0).

**Figure 8:**
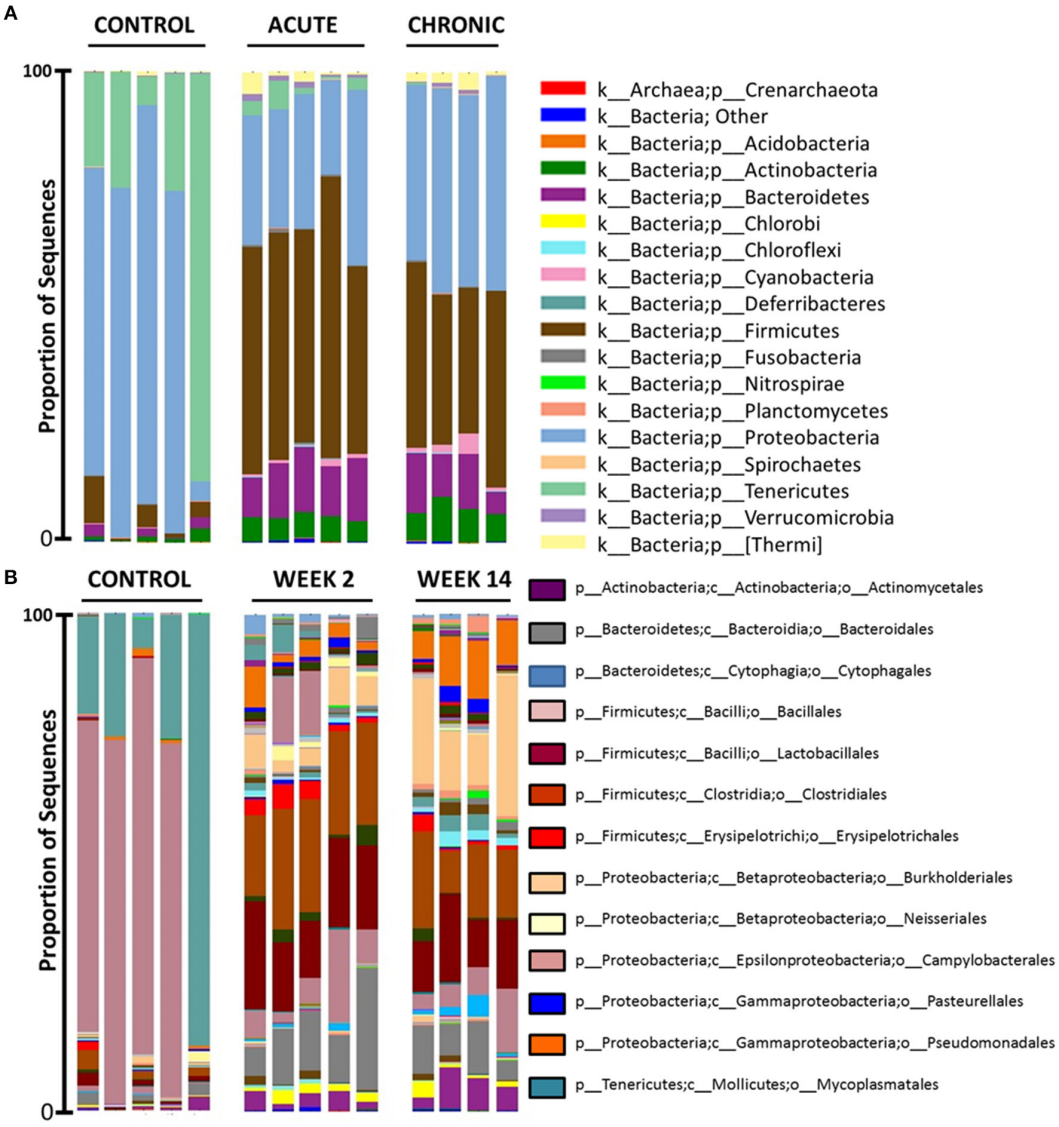
16s rRNA gene-based bacterial community composition of three groups [Control (*n* = 5), Week 2 (*n* = 5) and Week 14 (*n* = 4)]. Bacterial community sequence data are displayed at phylum **(A)** and order **(B)**.

At the bacterial phylum level, the middle meatus microbiota has relatively low diversity (Figure 8A). In control rabbits, members of *Proteobacteria* and *Tenericutes* dominated samples. Then on week 2, a shift of microbiota was observed and all five samples were dominated by members of *Firmicutes, Proteobacteria*, and *Bacteroidetes*. On week 14, bacterial community compositions were similar to those on week 2 but dominated by *Proteobacteria*, followed by *Firmicutes* and *Bacteroidetes* at phylum level: reduced *Firmicutes* and increased *Proteobacteria* compared to samples from rabbits on week 2. *Tenericutes* was significantly prevalent in controls compared to other sinusitis groups (uncorrected *p* = 0.003, FDR-*p* = 0.02). *Firmicutes* and *Bacteroidetes* were dominant both on week 2 and week 14 and significantly prevalent on week 2 (acute period) (*Firmicutes* uncorrected *p* = 0.003, FDR-*p* = 0.02; *Bacteroidetes* uncorrected *p* = 0.009, FDR-*p* = 0.03).

At the bacterial order level, members of *Campylobacterales* (from the phylum *Proteobacteria*) and *Mycoplasmatales* (from the phylum *Tenericutes*) dominated in the control rabbits (uncorrected *p* < 0.01, FDR-*p* < 0.05) (Figure 8B). On week 2, samples were dominated by mucin fermenting microbes (MFM): *Lactobacillales* (from the phylum *Firmicutes*), *Clostridiales* (from the phylum *Firmicutes*), and *Bacteroidales* (from the phylum *Bacteroidetes*) (uncorrected *p* < 0.01, FDR-*p* < 0.05). These rabbits displayed more variable microbiota than controls. On week 2, we noticed an emergence of *Burkholderiales* and *Pseudomonadales* and a decrease of *Campylobacterales* at the order level, compared to controls. On week 14, these rabbits exhibited increased *Burkholderiales* (uncorrected *p* = 0.003, FDR-*p* = 0.041) and *Pseudomonadales* (uncorrected *p* = 0.003, FDR-*p* = 0.041) and decreased *Lactobacillales* and *Clostridiales* (from the phylum *Firmicutes*), compared to samples from acute sinusitis rabbits on week 2.

In summary, MFM dominated on week 2 after blocking the sinus drainage pathway but was followed by a significant microbiome shift to potential pathogens (e.g., *Burkholderiales* and *Pseudomonadales*) during the development of chronic inflammation by week 14.

## Discussion

CRS is thought to persist as a consequence of chronic inflammation and remodeling that occurs with frequent and protracted episodes of ARS (Steinke and Borish, 2016). Once generated, CRS predisposes patients to recurrent and protracted bacterial infections with further damage to the epithelium, ciliary destruction, goblet cell metaplasia, prominent mucous gland and goblet cell hyperplasia, bacterial colonization, and biofilm formation, and ultimately leads to an inexorably worsening chronic inflammatory state (Payne et al., 2011; Steinke and Borish, 2016). Bacterial infection or colonization may play some role in initiating or sustaining the inflammatory response in CRS, but the study of dysbiosis of sinus microbiota in humans is especially challenging because any medical therapies can result in a dramatic change in the composition of resident bacterial communities, as observed in previous gut and sinus studies (Dethlefsen et al., 2008; Liu C. M. et al., 2013; Orlandi et al., 2016). Due to the heterogeneous nature of CRS, there have been marked differences in reported microbial communities between various studies. Therefore, there is a need for a preclinical model of sinusitis for longitudinal sampling to determine shifts in the sinus microbiota during the development of CRS.

In the current study, we created a rabbit model of sinusitis by blocking MCC of the maxillary sinus for 2 weeks (absence of infection), which resulted in the infiltration of sinus epithelium with acute inflammatory cells (neutrophils). When the packing was removed and the animals followed for another 12 weeks, rabbits had features of chronic inflammation (lymphocytes and plasma cells) on histology by week 14. Additionally, the functional microanatomy of airway epithelia *ex vivo* was found to have diminished mucociliary function with PCL depletion, decreased CBF, and severely delayed MCT compared to controls. These findings clearly establish the mechanistic basis for abnormal mucociliary host defense and point out the extent to which these changes to the epithelium can persist, despite resolution of the airway obstruction. They further indicate that PCL depletion can be an important mechanism in chronic sinus disease, as has been shown in the airways (Woodworth, 2015; Raju et al., 2017a,b; Tipirneni et al., 2017). Of note, PCL depletion can be a factor despite sufficient quantity of overlying mucus within the ASL. This may indicate the relevance of osmotically driven PCL depletion that occurs in the setting of high mucus concentration, a topic warranting further evaluation in this model. However, PCL depletion, decreased CBF, and delayed MCT also occurred at 14 weeks, but without mucus accumulation. With the development of the chronic stage, decreased bacterial clearance with the emergence of potential pathogens (e.g., *Pseudomonas*) may affect proper transepithelial ion transport and impair hydration of the ASL via multiple virulence factors [e.g., lipopolysaccharide (LPS), exotoxins] and result in acquired ion transport defects in the sinus epithelium (Cho et al., 2009; Raju et al., 2016; Solomon et al., 2016).

In this model, the sinus microbiota was also disturbed. The MFM dominated on week 2 after blocking the sinus drainage pathway followed by a significant microbiome shift during the development of chronic inflammation to potential pathogens (e.g., *Pseudomonas, Burkholderia*) by week 14. These data suggest that sinonasal dysbiosis (microbial imbalance) with anaerobic colonization or microenvironment may contribute to the pathogenesis of chronic sinus inflammation. Flynn et al. have shown that opportunistic pathogens which cannot degrade mucins (e.g., *P. aeruginosa*) do not become established in the airways until the MFM have colonized (Flynn et al., 2016). These MFM may contribute to chronic sinus disease by degrading mucins, which provide carbon-source nutrients for pathogens (non-fermenting bacteria) otherwise unable to obtain a carbon source in the sinus cavity. In the gastrointestinal tract, carbon-source nutrients (e.g., butyrate) produced by bacteria are thought to play an important and beneficial role (Consolandi et al., 2015). However, carbon-source nutrients that induce apoptosis in fibroblasts and lymphocytes (T- and B-cells) can be the toxic metabolic end product in the airway (Chang et al., 2013; Flynn et al., 2016; Sato et al., 2016). Despite the high density of bacteria that colonize the airway, nutrient sources that sustain bacterial growth *in vivo*, and how those nutrients are derived, are not well-characterized and the current rabbit model is well-suited to further elucidate such mechanism responsible for the generation of CRS secondary to pathogenic bacteria.

Several attempts have been made previously to create rhinosinusitis in animal models (Perez et al., 2014). Given the physiologic similarities between the rabbit and human sinonasal epithelium (Vaure and Liu, 2014), and previous development of an *in vivo* rabbit acute sinusitis disease model (Chiu et al., 2007), we selected the rabbit to create an animal model of CRS by endoscopically blocking the middle meatus without injuring the sinus mucosa. By targeting the middle meatus, our approach generated acute sinusitis in all (100%) rabbits on week 2, which was improved over other models where sponges were placed in the nasal cavity/floor (Liang et al., 2008). In studying the microbiome, no single animal model reproduces all aspects of human disease. However, phylogenetic and genetic comparisons of different mammalian genomes have clearly demonstrated that rabbits are closer to the human genome than rodents (Karlin et al., 1985). The rabbit microbiome is widely used to study human diseases, especially intestinal pathophysiology and periodontitis (Mapara et al., 2012; Jiminez et al., 2015). In the present study, the middle meati on week 14 were dominated by four major phyla—*Proteobacteria* (41.3%), *Firmicutes* (34.3%), *Bacteroidetes* (10.2%), *Actinobacteria* (7.5%), which is similar to human CRS (Jain et al., 2017; Wagner Mackenzie et al., 2017). Based on Wagner Mackenzie et al. (2017) only one genus *Corynebacterium* was identified as a potential biomarker of CRS-associated sinonasal microbiota and in this model, genus *Corynebacterium* (from the phylum *Actinobacteria*) was also significantly higher on week 14 compared to control and week 2 (*p* = 0.035). These data suggest the rabbit model provides a unique opportunity to disrupt, manipulate, and study sinus host–microbiome interplay at a level of experimental control that is not achievable in human studies.

In general, previous studies regarding the microbiome of human CRS identified less richness, evenness, and diversity compared to healthy controls as well as an increased burden of certain organisms (Wilson and Hamilos, 2014; Biswas et al., 2015). Conversely, the rabbit model exhibited higher bacterial diversity in acute and chronic sinusitis compared to the controls. It should be noted that increased microbial diversity has been identified in other diseases of the airway, such as chronic obstructive pulmonary disease (Pragman et al., 2012; Sze et al., 2012). There are two plausible explanations for these differences. First, bacterial dysbiosis in human CRS could result from multiple courses of prescribed antibiotics, thus contributing to lower bacterial diversity (Liu C. M. et al., 2013; Biswas et al., 2015). Several animal model studies have identified the increased severity of the inflammatory response when there was a reduced diversity of the stool microbiome induced by antibiotics (Maslowski et al., 2009; de Paiva et al., 2016). Another reason for decreased diversity is that in the late stage of chronic airway infection, host inflammatory responses and epithelial damage shape the micro-environment and increase the abundance of certain opportunistic pathogens (e.g., *Staphylococcus* or *Pseudomonas*) (Flynn et al., 2016). For example, previous studies have demonstrated that increased *Staphylococcus aureus* carriage in human CRS is associated with reduced bacterial diversity in human CRS (Feazel et al., 2012; Jain et al., 2017). Our rabbit model showed increased potential pathogens (*Burkholderiales* and *Pseudomonadales*) on week 14 compared to week 2. Once these opportunistic pathogenic bacteria form a niche (e.g., biofilm formation) in the late stages of CRS development (recalcitrant), we may observe decreased diversity after longer follow-up in rabbits. Perhaps pathogenic considerations regarding the microbiome should focus on community characteristics, which are largely governed by composition, and interactions among the constituent members, rather than diversity (Cho and Blaser, 2012).

Despite a surge in recent studies (Feazel et al., 2012; Biswas et al., 2015; Jain et al., 2017) that reveal a complex composition and dynamic changes in sinus microbiota in CRS, there is limited understanding regarding how microbes adapt to the airway microenvironment and which bacteria and pathways represent therapeutic targets. This current study underscores the importance of the depletion of PCL and the microbial ecological dynamics that contribute to the development of chronic sinusitis. Future studies examining the underlying mechanism are planned as such information will be crucial for identifying new therapeutic strategies for CRS management.

Limitations of the current study include relatively small sample sizes with only two-time points: Week 2 and Week 14. Well-controlled microbiota studies using animal models can show inter-study variations due to confounding factors in the experimental setup, such as animal origin, maternal effects, and environmental conditions (food composition, light, stress factors). However, the advantages of an animal model are numerous and the rabbit model created here allows control of multiple variables that are inherent to human samples, including genetics, medical history, environmental allergies, medication use, and exposure to environmental pollutants.

## Conclusion

We anticipate this reproducible model will provide a means for identifying underlying mechanism of ASL depletion and fundamental changes in sinus microbial communities that contribute to the development of CRS. The rabbit model of sinusitis exhibited diminished PCL depth with delayed mucus transport and significant alterations and shift in the sinus microbiome during the development of chronic inflammation.

## Author note

A part of this manuscript was presented at the COSM-American Rhinologic Society Spring Meeting, San Diego, CA, April, 2017.

## Author contributions

D-YC: designed the study, carried out the experiments and took the lead in writing the manuscript with support from SR, WS, and BW; CM and DS: carried out the rabbit and microbiome experiments and contributed to sample preparation; DS: analyzed the microOCT data; TS: contributed to the interpretation of the histology findings; WV: performed the analytic calculation microbiome data including diversity and PCoA plots with support from CM; SR and GT: verified the microOCT methods and helped supervise the microOCT analysis. All authors provided critical feedback and helped shape the research, analysis, and manuscript.

### Conflict of interest statement

D-YC receives research grant support from Bionorica Inc. BW is a consultant for Olympus and Cook Medical. He also receives grant support from Bionorica Inc. and Cook Medical. The other authors declare that the research was conducted in the absence of any commercial or financial relationships that could be construed as a potential conflict of interest.
